# Perturbations in the Urinary Exosome in Transplant Rejection

**DOI:** 10.3389/fmed.2014.00057

**Published:** 2015-01-05

**Authors:** Tara K. Sigdel, Yolanda W. Ng, Sangho Lee, Carrie D. Nicora, Wei-Jun Qian, Richard D. Smith, David G. Camp, Minnie M. Sarwal

**Affiliations:** ^1^Division of Transplant Surgery, Department of Surgery, University of California San Francisco, San Francisco, CA, USA; ^2^Department of Nephrology, Kyung Hee University, Seoul, South Korea; ^3^Biological Sciences Division, Pacific Northwest National Laboratory, Richland, WA, USA

**Keywords:** urine exosomes, kidney transplant, acute renal allograft rejection, biomarkers, proteomics

## Abstract

**Background:** Urine exosomes are small vesicles exocytosed into the urine by all renal epithelial cell types under normal physiologic and disease states. Urine exosomal proteins may mirror disease specific proteome perturbations in kidney injury. Analysis methodologies for the exosomal fraction of the urinary proteome were developed for comparing the urinary exosomal fraction versus unfractionated proteome for biomarker discovery.

**Methods:** Urine exosomes were isolated by centrifugal filtration of urine samples collected from kidney transplant patients with and without acute rejection (AR), which were biopsy matched. The proteomes of unfractionated whole urine (Uw) and urine exosomes (Ue) underwent mass spectroscopy-based quantitative proteomics analysis. The proteome data were analyzed for significant differential protein abundances in AR.

**Results:** A total of 1018 proteins were identified in Uw and 349 proteins in Ue. Two hundred seventy-nine overlapped between the two urinary compartments and 70 proteins were unique to the Ue compartment. Of 349 exosomal proteins identified from transplant patients, 220 had not been previously identified in the normal Ue fraction. Eleven Ue proteins, functionally involved in an inflammatory and stress response, were more abundant in urine samples from patients with AR, three of which are exclusive to the Ue fraction. Ue AR-specific biomarkers (1) were also detected in Uw, but since they were observed at significantly lower abundances in Uw, they were not significant for AR in Uw.

**Conclusion:** A rapid urinary exosome isolation method and quantitative measurement of enriched Ue proteins was applied. Perturbed proteins in the exosomal compartment of urine collected from kidney transplant patients were specific to inflammatory responses, and were not observed in the Ue fraction from normal healthy subjects. Ue-specific protein alterations in renal disease provide potential mechanistic insights and offer a unique panel of sensitive biomarkers for monitoring AR.

## Introduction

Exosomes are 50–90 nm vesicles secreted by a wide range of mammalian cell types as a consequence of fusion of multivesicular late endosomes/lysosomes with the plasma membrane (2, 3). During the construction of endosomes and intraluminal vesicles (ILVs), specific membrane, and cytosolic proteins are incorporated and secreted in the excretory pathway of exosomes. Therefore, exosomes from different cellular origins contain cell-type specific components that mirror the biological function of the parent cell as well as common molecules, which are needed for their structure and function (4). Recent evidence suggests that exosomes are also involved in the modulation of immune function and dissemination of several infectious cargos such as HIV. Exosomes can also be involved in antigen presentation to T cells (5) and the development of tolerance (6). DC-derived exosomes called dexosomes express high levels of functional MHC class-I and class-II peptide complexes along with CD86 molecules (7). It was also suggested that exosomal RNA could be transferred between cells and represents a new mechanism of gene-based communication between mammalian cells (8). These immune modulating and cell to cell communication properties of exosomes indicate that urine exosomes could be an advantageous source to study the mechanisms of and discover useful biomarkers in variable kidney diseases, including the study of various injury processes in kidney transplantation.

The evolution of current mass spectrometry (MS)-based proteomic approaches have contributed substantially to our understanding of the molecular characterization of urine proteins. However, one of the main hurdles for biomarker discovery with urine proteomics is discerning proteins present at low levels from other highly abundant proteins such as albumin and uromodulin. In addition, the heterogeneity of urine protein added its complexity in urine proteomics. The origin of protein in proteinuric patients could be one of the following; first, the filtrate of plasma protein through intact (overflow proteinuria) or damaged glomerular basement membrane or podocyte (glomerulopathy), finally, excretory vesicles (exosomes) or membrane-shed vesicles (microparticles, also referred to as ectosomes) from kidney and uroepithelial cells. In contrast to established proteomic approaches, subproteome approaches which direct to study certain excretory vesicles such as exosomes could be more informative for disease identification and progression, because it could offer the simple way to get rid of the most abundant protein from the unprocessed urine and simplify the source of urine protein, especially for the proteinuric patients.

Amid the early experiments to evaluate the route for the tubular protein in urine, Pisitikun et al. isolated the exosomes from urine with a differential ultracentrifugation method and confirmed the expression of several apical transporter proteins, which originated from renal tubules. More recently, this group analyzed the urine exosomes using liquid chromatography coupled with tandem mass spectrometry (LC-MS/MS) and identified 1132 proteins (2). Other studies to find the biomarkers from specific small vesicles for prostate and bladder cancers were also attempted (3, 4). Miranda et al. also showed human urinary exosomes contained enough mRNA for kidney specific genes (5). Nevertheless, the absence of a standardized protocol for isolating exosomes from clinical specimens and adequate normalization for timed collections are hurdles to the clinical application of urine exosomes. However, recent approaches, which use nanomembrane and microfilter centrifugation to isolate exosome enriched fractions, suggested that urine exosomes could be a reliable source for biomarker discovery and incorporated into the clinical application (6, 7). We tested a centrifugal filtration method to isolate exosomes from the urine of kidney transplant recipients. Our result demonstrated that the centrifugal filtration has advantage over the ultracentrifugal method in its simple experimental setup, less time spent in exosomal extraction, and identification of novel exosomal proteins that were not reported by previously published works.

Data are lacking on the regulation of exosomes in physiologic stress and what differences occur in the urine exosomal fraction under various pathologic conditions in kidney transplantation. If the phenotype specific differences could be identified in exosomes from the patients with different transplant-associated renal injuries, subproteomics using exosomes could serve as good complement to current proteomics and genomic approaches for improving on our knowledge on the mechanism and monitoring of renal injury.

In contrast to established proteomic approaches, subproteome approaches which direct to study certain excretory vesicles such as exosomes could be more informative for disease identification and progression, because it could offer a method for biomarker discovery in renal diseases without the confounding influence of abundant proteins found in unprocessed urine.

In this study, we have undertaken a pilot study of 30 urinary samples from renal transplant patients with biopsy proven AR and 20 urine samples from non-AR controls with other forms of renal injuries such as BK nephropathy (BKVN) and chronic allograft injury (CAI). The purpose of the study was to study if phenotype specific differences could be identified in exosomal enrichment fraction in urine from patients specific to biopsy confirmed acute renal transplant rejection.

## Materials and Methods

### Study population and samples

A total of 30 mid-stream, second morning void urine samples from kidney transplant patients that included non-acute rejection (nAR; *n* = 20) and acute rejection (AR; *n* = 10) were selected from a large and highly annotated biobank of urine samples collected from pediatric and young adult recipients of kidney transplants from 2000 to 2009 at Lucile Packard Children’s Hospital at Stanford University. The bio-repository consisted of ~2000 banked urine samples of which 770 were biopsy matched and collected prior to any treatment intensification for clinical graft dysfunction. “Allograft injury” in this study was defined as a >20% increase in serum creatinine from its previous steady-state baseline value and an associated biopsy that was pathological. All biopsies were blindly semi-quantitatively scored by a single pathologist using the most recent Banff criteria for both acute and chronic injury (1, 8–10). AR was defined at minimum, as per Banff Schema, a tubulitis score ≥1 accompanied with an interstitial inflammation score ≥1, nAR patients were inclusive of patients with CAI and was defined at minimum, as tubular atrophy score ≥1 accompanied by an interstitial fibrosis score ≥1. Also, included were BKVN that was defined by a demonstration of a positive blood BK viral load, graft inflammation, and a positive immunohistochemical stain for the polyoma virus. The patients included in this study were all on a maintenance combination of tacrolimus, mycophenolic acid, and were either on maintenance steroids or on a steroid avoidance protocol (11). The study was approved by the Ethics Committee of Stanford University Medical School and University of California San Francisco, and all patients/guardians provided informed consent to participate in the research, in full adherence to the Declaration of Helsinki. This study was approved by the Institutional Review Board for Human Subjects Research at Pacific Northwest National Laboratory in accordance with federal regulations.

### Urine collection, initial processing, and storage

We used standards recommended by human kidney and urine proteome project (HKUPP) while collecting and processing samples that were applicable in the kidney transplant clinic. Second morning, void, mid-stream urine samples (50–100 mL) were collected in sterile containers and centrifuged at 2000 × *g* for 20 min at room temperature within 1 h of collection. The supernatant was separated from the pellet containing any particulate matter including cells and cell debris. The pH of the supernatant was adjusted to 7.0 and stored at −80°C until further analysis. Prior to these proposed studies, we established protocols that allowed for stable urine collection from multicenter clinical studies (12), where delays in storage and processing can occur. With our protocols, urine samples can be safely stored up to 1 h at room temperature and up to 12 h at 4°C without significant protein degradation; samples do not require addition of protease inhibitors to improve sample integrity if stored at 4°C or −80°C within 72 h; and centrifugal filtration was our optimal processing method. In order to ensure minimum impact of freeze thaw cycles, we aliquoted urine samples into 10 mL aliquots (5–10 tubes per sample) prior to freezing, to ensure that multiple assays can be done without multiple freeze thaw cycles. Our assay utilized 10 mL starting urine so each aliquot only needed to be thawed once for the experiments.

### Isolation of protein from whole urine

We followed previously published method that was developed in the lab for urine protein isolation (13). Briefly, proteins were isolated by using centrifugal filtration of the supernatant through Amicon Ultra centrifugal filtration tubes (10,000 molecular weight cutoff, Millipore, Bedford, MA, USA). The filter tube was initially washed with 10 mL of 50 mM NH_4_HCO_3_ (pH 8.0) and discarded. Then a 10 mL aliquot of urine was loaded into the device and centrifuged for 20 min at 3000 × *g* at 10°C, and the retentate was used as protein extract for the whole urine.

### Isolation of urine exosomes

#### Exosome isolation by ultracentrifugation

Clarified urine (10 mL) was centrifuged at 200,000 × *g* in a fixed angle rotor (45Ti Beckman Instruments) for 110 min. The supernatant was removed and the pellet washed with a large volume of 1× phosphate buffered saline (PBS) and centrifuged again at 200,000 × *g* for 110 min. The pellet was re-suspended in isolation buffer (10 mM triethanolamine, 250 mM sucrose, pH 7.6) supplemented with protease inhibitors (Complete Mini) and protein concentration determined using a microBCA assay (Pierce).

#### Exosome isolation by nanomembrane concentrator

A 10 mL volume of urine was thawed and 12.5 μL of Protease Inhibitor Cocktail (Sigma-Aldrich P2714; prepared by adding one vial to 5 mL nanopure water) per mL of urine was added. First, a pool of urine samples was prepared by adding 0.5 mg urine creatinine equivalent of urine (average urine volume 7.0 ± 2.3 mL) to each pool of AR, BK, and CAI. An equal volume of 1× PBS buffer was added to the urine. The urine was centrifuged at 2500 × *g* for 15 min at 25°C and transferred to high-speed tubes and then centrifuged at 17,000 × *g* for 30 min at 25°C. The supernatant was transferred to a PBS buffer equilibrated nanomembrane concentrator (Vivaspin 20-PES 100,000 MWCO; VS2041) and centrifuged at 3000 × *g* at 25°C for 30 min. The filtrate was saved for separate analysis. The retentate was washed with 20 mL of PBS by centrifuging at 3000 × *g* at 25°C for 20 min. The volume of the retentate was adjusted to 200 μL for downstream proteomic analysis.

### One-dimensional, denaturing, reducing electrophoresis, and immunoblotting

Exosomal proteins (5–20 μg) were separated using SDS-PAGE on 4–12% NuPAGE gels (Invitrogen) at 200 V until the bromophenol blue running dye migrated to the end of the gel using Mark12 molecular weight standards (Life Technologies, Carlsbad, CA, USA) and HK2 cell lysates or total urine protein as positive controls when possible. Proteins were electro-blotted and probed using previously published methods (14). Primary antibody (0.2–1.0 μg/mL) and secondary antibody (0.05–0.2 μg/mL) were dissolved in 5% w/v bovine serum albumin (Sigma-Aldrich) in TTBS (Sigma-Aldrich). Secondary antibody conjugated with horse-radish peroxidase (HRP) was visualized using the Pierce SuperSignal^®^ West Pico Chemiluminescent Substrate Kit. Membranes were used to expose x-ray film and resulting images were developed, scanned, and bands quantified.

### Transmission electron microscopy of whole-mounted exosomes

Exosome samples recovered in PBS were used for TEM characterization. Exosomes were pelleted by high-speed ultracentrifugation at 200,000 × *g*. PBS was removed by pipetting and 4% glutaraldehyde (Polysciences, Warrington, PA, USA) in PBS was layered onto the exosomal pellet. Exosomal-protein glutaraldehyde-cross-linking was allowed to proceed for 1 h. The cross-linked pellet was submitted for TEM analysis. The cross-linked exosomes were dehydrated in graded ethanol and flat-embedded in LX-112 epoxy resin (Ladd Industries, Burlington, VT, USA). Selected areas were mounted on blocks, ultra-thin sections (70–80 nm, silver-gray interference color) were cut using a diamond knife (Diatome, Fort Washington, PA, USA), and sections collected on copper grids. The sections were stained with saturated solutions of uranyl acetate, rinsed and submitted for imaging using a Philips CM10 transmission electron microscope operating at 60 kV.

### Quantitative urine exosomal proteomics by using iTRAQ and LC MS/MS

All peptide samples were assayed with bicinchoninic acid (BCA) (Thermo Scientific, Rockford, IL, USA) to determine the protein concentration. An equal mass of protein (20 μg) was collected from each sample and brought to a volume of 30 μL using 0.5 M triethylammonium bicarbonate (TEAB) (all chemicals purchased from Sigma-Aldrich, St. Louis, MO, USA, unless otherwise stated). 2,2,2-Trifluoroethanol was added to the samples for a final concentration of 50% TFE. The samples were sonicated in an ice-water bath for 1 min and incubated at 60°C for 2 h with gentle shaking at 300 rpm. The samples were then reduced with 2 mM dithiothreitol (DTT) with incubation at 37°C for 1 h with gentle shaking at 300 rpm. Iodoacetamide was added to reach a final concentration of 40 mM for alkylation and incubated in the dark at 37°C for 1 h with constant shaking. The samples were then diluted sevenfold using 0.5 M TEAB and digested with trypsin [50:1 protein:trypsin (w/w)] (Promega, Madison, WI, USA) at 37°C for 3 h. To clean the peptides, C-18 solid phase extraction (SPE) was performed using Supelco Discovery columns with a Gilson GX-274 ASPEC™ system (Gilson Inc., Middleton, WI, USA) and eluted into a low-protein binding 1.5 mL centrifuge tube. All samples were then dried in a speed-vac to 15 μL and assayed with BCA to determine the peptide concentration.

#### 8-plex iTRAQ labeling

The pH of each sample was measured and brought to ~pH 8 using 1 M TEAB. Each vial of 8-plex iTRAQ reagent (AB Sciex, Framingham, MA, USA) was brought to room temperature. The reagents were pulse spun to ensure the contents were collected at the bottom and 60 μL of isoproponal was added to each reagent vial. The reagents were thoroughly vortexed, spun down, and added to the appropriate sample. The reagent vials were rinsed with an additional 10 μL of isopropanol and added to the samples.

The iTRAQ reagents with 8 channels 114–121 were used to label 2 pooled AR samples with each split into 2 as duplicates, 2 BKV, and 2 CAI, respectively, for each 8-plex iTRAQ experiment. Three 8-plex experiments were performed for the 24 pools from the 4 phenotypes. For the labeling reaction, the pH was above 7.8 and the organic concentration was at least 60% (v/v). Each sample was vortexed and spun down to incubate at room temperature for 2 h at which time 100 μL of nanopure water were added to hydrolyze the sample and incubated for an additional 30 min. The samples were partially dried down in a speed-vac to remove the organic solvent and then pooled together and dried down to a volume of ~100 μL. An SPE C18 OMIX tip (Agilent, Santa Clara, CA, USA) was used to clean the final sample for 2D-LC-MS/MS analysis.

#### 2D-LC-MS/MS analysis

The final 8-plex iTRAQ-labeled sample was subjected to 2D-LC- MS/MS analysis (15). The 2D-LC system was custom-built using two Agilent 1200 nanoflow pumps and one 1200 capillary pump (Agilent Technologies). Use of dual trapping and reversed-phase columns allowed for parallel event coordination, allowing for fraction trapping and washing offline while analytical separation occurred on the other reversed-phase column. Columns were manufactured in-house by slurry packing media into fused silica (Polymicro Technologies Inc.) using a 1 cm sol-gel frit for media retention. First-dimension SCX column: 5 μm PolySULFOETHYL-A (PolyLC Inc.), 15 cm × 360 μm od × 150 μm id. Trapping columns: 5 μm Jupiter C18 (Phenomenex), 4 cm × 360 μm od × 150 μm id. Second-dimension reversed-phase columns: 3 μm Jupiter C18 (Phenomenex), 35 cm × 360 μm od × 75 μm id. Mobile phases consisted of 0.1 mM NaH_2_PO_4_ (A) and 0.3 M NaH_2_PO_4_ (B) for the first-dimension column and 0.1% formic acid in water (A) and 0.1% formic acid ACN (B) for the second-dimension column. The SCX separation provided 15 fractions for the second-dimension reversed-phase gradient nanoLC-MS/MS with 20 μL for each fraction.

Mass spectrometry analysis was performed on a Thermo Fisher Scientific LTQ-Orbitrap Velos MS (San Jose, CA, USA) using a 150 μm od × 20 μm id chemically etched fused-silica electrospray emitter (16). The SCX separation for 15 fractions was performed stepwise, each step taking 20 min. Each fraction was trapped on C18 material and washed for 50 min using the second-dimension mobile phase A. The gradient was then started and 15 min later acquisition was started and continued for 100 min for each fraction. The heated capillary temperature and spray voltage were 350°C and 2.2 kV, respectively. Full MS spectra were recorded at a resolution of 30,000 (for ions at *m*/*z* 400) over the range of *m*/*z* 400–2000 with an automated gain control (AGC) value of 1e6. MS/MS was performed in the data-dependent mode with an AGC target value of 3e4. The ten most abundant parent ions, excluding single charge states, were selected for MS/MS using high-energy collisional dissociation (HCD) with a normalized collision energy setting of 40%. A dynamic exclusion time of 45 s was used.

### Statistical analysis

A fold change over the global normal was calculated for each protein in the soluble and exosomal fractions. Two tailed unpaired *t*-tests were performed with SPSS software to determine the significance of differences in fold change over global normal between Uw and Ue compartments and differences in fold change over the global normal between AR and no AR in the exosomal fraction.

## Results

### Patient demographics

A total of 30 urine samples were collected from AR (*n* = 10) and nAR (*n* = 20) patients. In the AR population, at time of sample collection, age ranged from 1.9 to 19.4 years of age (mean = 14.5 years), 40% were female, 70% received deceased donor transplants, 30% were living related donors, serum creatinine ranged from 0.2 to 1.8 mg/dL (mean = 1.15 mg/dL) with a calculated creatinine clearance (by the Schwartz equation) of 63–206 mL/min/1.73 m^2^ (mean = 95 mL/min/1.73 m2). Primary kidney diseases included focal segmental glomerulosclerosis (FSGS, 30%), reflux nephropathy/obstructive uropathy (20%), dysplasia (10%), Alport’s syndrome (10%), lupus nephritis (10%), and 20% were unknown. In the nAR population, at time of sample population, age ranged from 1.7 to 18.7 years of age (mean = 12.1 years) 30% were female, 50% were deceased donors, and 50% were living related donor transplants, serum creatinine at time of collection ranged between 0.3 and 3.5 mg/dL (mean = 1.2 mg/dL) with a calculated creatinine clearance range of 20–167 mL/min/1.73 m^2^ (mean = 89 mL/min/1.73 m2). Primary kidney diseases included reflux nephropathy/obstructive uropathy (25%), unknown (20%), FSGS (10%), 1 patient had both reflux and FSGS, dysplasia (10%), vasculitis (10%), dysplasia (10%), nephrotic syndrome (10%), and the remainder with, Alport’s syndrome, cortical necrosis, and non-focal glomerulosclerosis.

### Urine exosome isolation by centrifugal filtration

**Figure 1:**
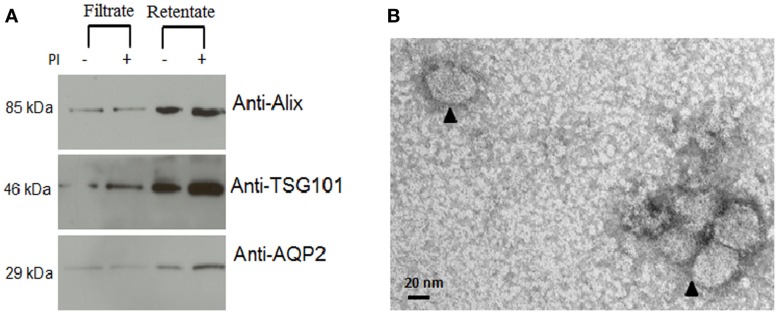
**Confirmation of isolation of exosomal vesicles from human urine**. **(A)** Western blot demonstrated significant enrichment of exosomal marker proteins in the retentate fraction. From the image analysis, there was more retention of intact protein in the urine with added protease inhibitors than without added protease inhibitors. **(B)** Electron micrograph (EM) of isolated exosomes in the exosome extract.

Several exosomal extraction methods have been reported previously and included ultracentrifugation (2, 17, 18), nanomembrane concentrator (6), and immunoisolation (19, 20). We tested two methods that used ultracentrifugation and nanomembrane concentrator for this study. When we used 10 mL urine from three different urine samples, the ultracentrifugation method did not provide sufficient exosome protein extract (16 ± 9 μg/mL raw urine) for Western blot analysis (data not shown). Applying the nanomembrane concentrator method to isolate urine exosomes, did not require ultracentrifugation and provided much better exosomal yield in terms of total protein (678 ± 93 μg/mL raw urine) from the same set of three urine samples. The 100 kDa centrifugal concentrators were used to isolate urine exosomes that could be analyzed to verify for exosomal extract by Western blot and electron microscopy (Figure 1). Programed Cell Death 6 Interacting Protein (ALIX), Tumor Susceptibility 101 (TSG101), and Aquaporin (AQP) were selected as markers for exosome vesicles (17, 21) for the Western blot. The enriched exosomal extract was tested for both the filtrate and the retentate fraction from the concentrator (Figure 1A). As demonstrated by electron micrograph (EM) in Figure 1B, the exosomal extract contained exosomal vesicles. This method was performed with urine at room temperature that helped in minimizing the formation of Tamm–Horsfall protein aggregates that have been reported to be a major challenge when using ultracentrifugation which needs to be carried out at 4° C (22).

### Detection of novel exosomal proteins in acute rejection: Expansion of the urine exosome database

**Figure 2:**
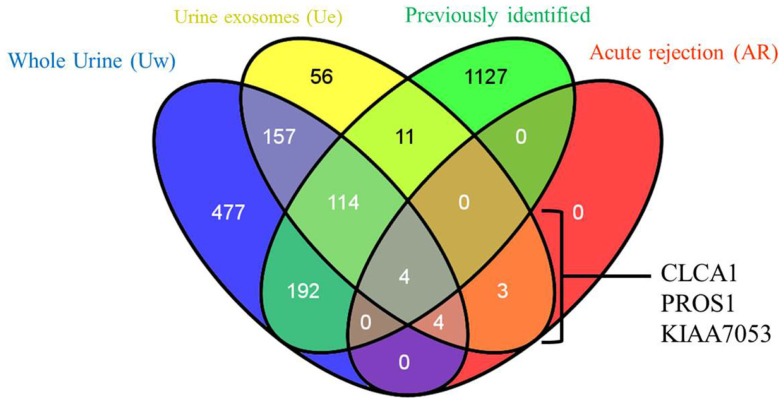
**Relationship of total urine exosomal proteins (Ue), urine exosomal proteins specific to acute rejection (AR) when compared to soluble proteins in whole urine (Uw) and urine exosomal proteins previously published in literature (previously identified)**. We identified 59 novel urinary exosomal proteins that are exclusive to the exosomal fraction, including 3 (CLCA1, PROS1, and KIAA053) of which demonstrated an increase fold change in the AR samples only. Venn diagram created with *VENNY* (25).

**Figure 3:**
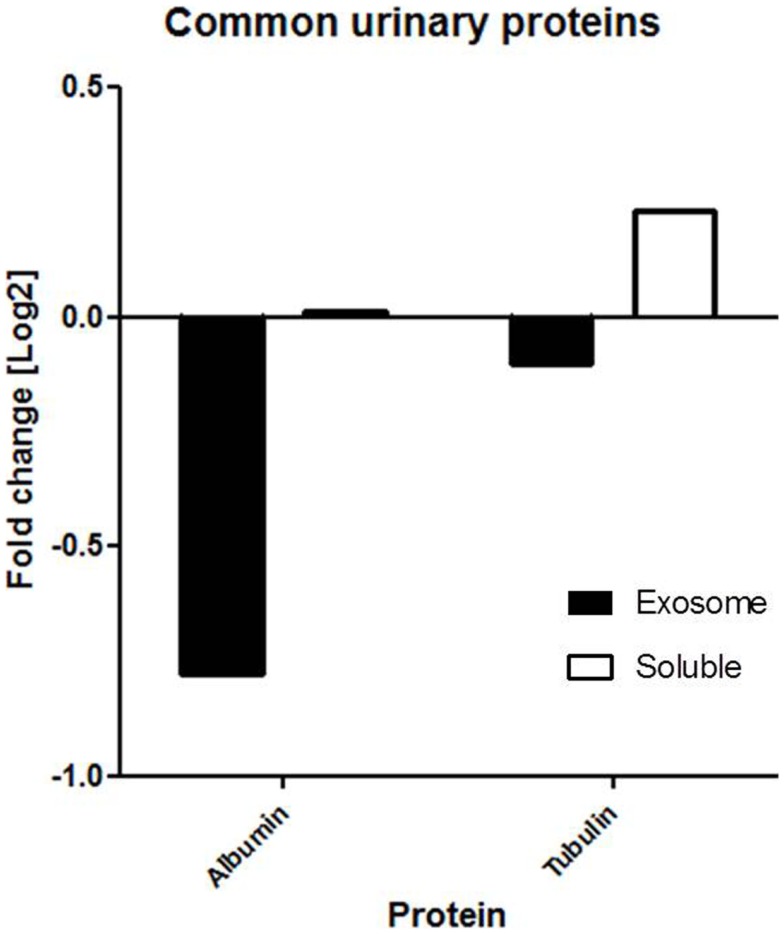
**Common proteins (e.g., albumin and tubulin) are not found in abundance in the exosomal fraction**. Our findings show that the fold change of albumin and tubulin are much lower in the exosomal fraction than soluble fraction of urine.

2D-LC-MS/MS analyses of urine exosome samples resulted in the identification of a total of 349 proteins in the exosomal extract of which 343 proteins were identified in all injury phenotypes (Table S1 in Supplementary Material). We performed gene ontology (GO) analysis which revealed that these proteins were enriched in a number of biological pathways such as platelet degranulation (*p*-value = 8.07e-4) and activation (*p*-value = 7.39e-3), humoral immune response (*p*-value = 1.099e-9), complement activation (*p*-value = 2.410–9), and response to stress (*p*-value = 1.06e-4). GO molecular functions enriched are lipid transport (*p*-value = 1.979e-3), lipoprotein binding (*p*-value = 3.579e-4), and antioxidant activity (*p*-value = 5.049e-3). The cellular components enriched included blood extracellular components (*p*-value = 2.820e-21), cell surface components (*p*-value = 1.578e-8), blood mircoparticle (*p*-value = 2.769e-8), secretory granule lumen (*p*-value = 4.520e-8), and cytoplasmic membrane-bounded vesicle lumen (*p*-value = 7.849e-8). Evaluation of public domain data for urine proteins previously described in the Ue fraction of healthy subject control urine in both the ExoCarta database and published literature (14, 23) revealed that 220/349 Ue proteins in kidney transplant patients had not been previously identified in the healthy Ue fraction, indicative of unique Ue proteins in kidney transplant recipients. When we compared the 349 proteins to those previously identified in the whole urine proteome data set (13), 70 are unique to the exosomal fraction and 59 of these exclusive Ue proteins had not been previously identified (Figure 2). They mainly enriched for biological pathways related to cell mediated immunity and response to stress. The most significant molecular function was structural molecule activity and their cellular components were mainly extracellular or vesicle components. Twenty-five percent (18) of the proteins exclusive to the exosomal fraction were enriched for their higher expression in the renal cortex, based on our previous studies (24). High abundance urinary proteins, such as albumin and tubulin, were not enriched in high abundance in the Ue fraction (Figure 3).

### Identification of AR-specific urine exosomal proteins

**Figure 4:**
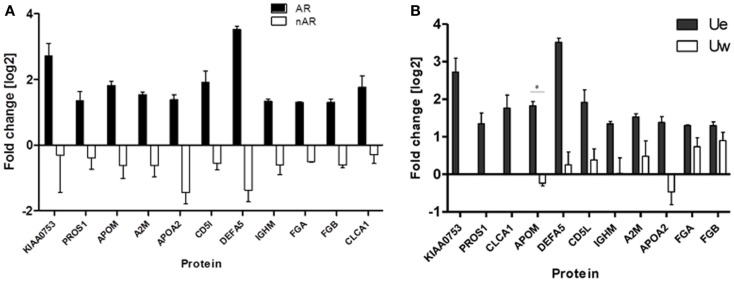
**(A)** Urine from patients in the acute rejection population enriched for eleven exosomal proteins with an increased fold change over the whole urine fraction. All, except CLCA1, have significantly (*p*-values <0.05) increased fold change in the Ue of AR when compared to Ue of nAR. **(B)** They are also enriched in Ue over Uw. CLCA1, PROS1, and KIAA0753 were detected in the exosomal fraction only, however, there is only significantly increased enrichment (*) was observed for ApoM (*p* = 0.02).

We quantitatively compared the abundance of the exosomal proteins in the AR urine to the exosomal proteins in the nAR urine using iTRAQ reagents. Eleven proteins namely Alpha-2-macroglobulin (A2M), Apolipoprotein A-II (APOA2), Apolipoprotein M (APOM), CD5 antigen-like (CD5L), calcium-activated chloride channel regulator-1 (CLCA1), fibrinogen alpha chain (FGA), fibrinogen beta chain (FGB), immunoglobulin-mu chain C region (IGHM), defensin-5 (DEFA5), vitamin K-dependent protein S (PROS1), and protein KIAA0753(KIAA0753) were found to be increased in the exosomal fraction and significantly increased in the AR population (Figure 4; Table 1). Three of these proteins, calcium-activated chloride channel regulator 1, Protein S, and KIAA0753 were found exclusively in the exosomal fraction. They enriched for GO biological pathways such as platelet degranulation (*p*-value = 1.130e-8) and processes related to lipoprotein assembly and clearance (*p*-value = 1.130e-8). We performed two tailed *t*-tests of these proteins between Ue and Uw as well as AR and nAR. In the AR and nAR comparisons, DEFA5, a protein involved in the innate immune response, had the greatest significant increase in AR (4.89 ± 0.23, *p* = 3.3e-5), however, the difference in fold change in the Ue versus the Uw compartment was not significant. CLCA1 had the least significant increase in AR (*p* = 0.052). Six urine exosomal proteins were previously unidentified. APOM is a component of kidney epithelial cells and has been previously linked to renal injury (26) but had not been previously identified as a urine exosomal protein. In our studies, we found it to be significantly increased in the exosomal fraction (*p* = 0.022, CI = 1.15–2.88) and significantly increased in the AR population (*p* = 0.001, CI = 1.64–3.22).Three novel urine exosomal proteins, CLCA1, PROS1, and KIAA0753, were exclusively found in the AR population of renal transplants. CLCA1 is a 100 kDa secreted membrane associated human protein typically found in mucus producing goblet cells. It is involved in mediating calcium-activated chloride conductance (27). There is evidence that it is involved in the regulation of tissue inflammation in the innate immune response (28). PROS1 codes for the 75 kDa secreted human Protein S anticoagulant, which is a co-factor to activated protein C and is involved in the regulation of the coagulation cascade by degrading Factors Va and VIIIa. Thus, it is involved in blood coagulation and is associated with extracellular vesicles. Based on information from GeneCard, KIAA0753 encodes for a protein involved in ubiquination and protein degradation. Eight Ue AR-specific markers are detected in Uw, but as they are at much lower abundance levels in Uw, they are not significant for AR in Uw.

**Table 1:** **AR exosomal proteins**.

Gene ID	Description	Function	GO biological processes	GO cellular components	Average fold change: (nd, not detected)			Fold change: Ue (AR)-Ue (nAR)	
					Ue	Uw	*p*	Δ	*p*
DEFA5	Defensin, alpha 5, Paneth cell-specific	Antimicrobial activity possibly via membrane permeabilization	Antibacterial response; humoral and innate response	Extracellular; Golgi lumen; cytoplasmic vesicle	3.52	0.51	0.22	4.89	3.36e-05
KIAA0 753	109 kDa protein	Uncharacterized protein, associated with protein degradation	n/a	Cytoplasm; centrosome	2.50	nd	n/a	3.03	0.055
CD5L	CD5 molecule-like	Regulates immune system possibly via apoptosis	Apoptosis; cellular defense response	Extracellular	1.92	0.32	0.17	2.47	0.015
APOM	Apolipoprotein M	Binds various fatty acids, most likely involved in lipid transport	Cholesterol homeostasis, transport, and assembly; antioxidant activity; response to glucose	Extracellular; HDL/VLDL particles; plasma membrane	1.82	−0.19	0.02	2.43	0.001
CLCA1	Calcium-activated chloride channel regulator 1	May mediate calcium-activated chloride conductance; associated with immune response and inflammation	Calcium ion and chloride transport; cellular response to hypoxia	Extracellular; plasma membrane; microvillus	1.76	nd	n/a	2.06	0.052
A2M	Alpha-2-macroglobulin	Inhibits all 4 classes of proteases by trapping mechanism	Hemostasis/plate let activation; negative regulation of complement; stem cell differentiation	Cytosol; extracellular; platelet alpha granule lumen	1.53	0.48	0.08	2.16	0.001
APOA2	Apolipoprotein A-II	May stabilize HDL	Acute inflammatory response; cholesterol homeostasis, transport, and assembly; host virus interaction	Chylomicron; endoplasmic reticulum lumen; extracellular; HDL/VLDL particle	1.38	−0.47	0.28	2.83	0.001
PROS1	Vitamin K-dependent protein S	Activated protein C co-factor, degrades factors Va and VIIIa; stimulates fibrinolysis	Anticoagulation; leukocyte migration and immune response	Extracellular; Golgi membrane	1.35	nd	n/a	1.74	0.023
IGHM	Immunoglobulin heavy constant mu	Constant region of IgM, a soluble and membrane bound antibody	Immune response; antibacterial immune response	Extracellular; plasma membrane bound	1.34	0.22	0.41	1.95	0.006
FGA	Fibrinogen alpha chain	Component of fibrinogen for platelet aggregation	Coagulation; platelet activation and platelet degranulation; signal transduction	Extracellular; plasma membrane; extracellular vesicles; fibrinogen complex	1.3	0.73	0.06	1.81	0.006
FGB	Fibrinogen beta chain	Component of fibrinogen for platelet aggregation	Coagulation; platelet activation and degranulation; signal transduction		1.3	0.9	0.16	1.90	0.006

In this study, urinary neutrophil gelatinase-associated lipocalin (NGAL), which is a well-known urinary marker of kidney injury (29) and graft dysfunction (30), was not identified despite the fact that it has been reported to be detected in urine exosomes by ELISA (31) and also by us in the mass spectrometric analysis of soluble proteins in the urine (13). Another well-known urinary protein, kidney injury molecule (KIM1) (32) was not identified in this study but has been identified in the soluble urinary fraction of urine collected from kidney transplantation patients (data not shown). The absence of the well-known markers from our list of 349 urine exosomal proteins is attributed to the fact that their lower abundance in the urine and the dependence of unbiased identification by a quantitative MS method, such as iTRAQ, on protein abundance is biased toward proteins that are enriched in the study sample (urine exosome) in this case.

## Discussion

Because of the potential of exosomes containing enriched candidate biomarkers for diseases there is a growing interest in exosome isolation and investigation of its compositions. It has been shown that exosomes are shed in urine and contain membrane bound proteins and proteins involved in cell signaling, inflammation, and originate from renal epithelial cells (2, 23). Previous studies have shown that urine exosomes were enriched in innate immune modulators, but there have not been studies that describe the enrichment of exosomes in transplant injury compared to normal in solid organ transplantation. Our Uexo extraction and purification method is not only simple but is also readily transferrable to other clinical labs.

We observe an enrichment of exosome specific proteins in kidney transplant patients who have biopsy proven AR, and perturbation in the Ue fraction by disease resulted in a repertoire very different from the Ue fraction in normal health. In fact, only 9% of the Ue proteins detected in healthy urine were noted in the Ue fraction from patents with renal transplants, and most of these overlapping Ue proteins are involved in vesicle-mediated transport (*p* = 2.109e-14), regulatory mechanisms (2.109e-14), and response to stress (*p* = 1.060e-13). In kidney transplantation, the ischemic and alloimmune injury to the donor kidney resulted in marked changes in the Ue fraction, with an enrichment of proteins involved in inflammation, cell division, tissue repair, and the immune response. AR creates an environment of inflammation of the renal tubule (the tubulits injury in AR), interstitium, and the vascular space and the altered response of the injured tubules to these infiltrating cells is seen by a predominance of proteins involved in cell repair (*p* = 0.037) and fibrosis (*p* = 0.03).

The orchestrated injury and its local response in transplant rejection is highly specific and likely explains the almost complete alteration of the Ue fraction in urine samples taken from organ transplant patients versus normal healthy volunteers. The highly specific nature of the perturbations of the Ue compartment in disease, make the study of this space very attractive in terms of studying disease and injury mechanism. Additionally, as these altered protein fractions mirror the local organ injury, monitoring for changes in the abundance of these proteins could be tracked as sensitive disease and injury specific biomarkers. Our studies indicate that the Ue-specific AR biomarkers in this pilot study are highly specific for AR versus other types of transplant injury, and can only be identified by Ue analysis due to their very low abundance or even complete absence from similar analysis of unfractionated whole urine.

Within this compartment, we identified proteins (CLCA1, PROS1, and KIAA0753) that are exclusive to AR patients and have not been previously identified in the available databases of healthy urine exosomal proteins. Since these proteins are enriched in inflammatory responses, they may serve as valuable useful markers of rejection. Also, within this compartment is ApoM, which has been previously identified to in soluble urine and associated with kidney injury. We show that exosomal ApoM is more abundant than soluble ApoM thus supporting that exosomal proteins could serve as a more sensitive biomarker. Numerous studies, including those originating from our group have shown that using elevated creatinine and biopsy results to identify renal transplant dysfunction and rejection is often a late finding (33–36). Further studies are needed to evaluate if the Ue-specific proteins in AR, are altered earlier in the course of rejection injury, and if their altered detection, when altered, can predate a rise in the serum creatinine providing the potential for early intervention and prompt injury reversal.

In conclusion, in this report, we have optimized a rapid urinary exosome isolation method and quantitative measurement of enriched Ue proteins. Our observation from this study demonstrated that proteins in the exosomal compartment of urine collected from kidney transplant patients were specific to inflammatory responses, which were different from normal healthy subjects. However, the small sample size of the study and because of the possibility of >100 kDa proteins isolation due to the cutoff size of the nanomembrane concentrater, future studies with larger sample size and further refinement in the exosomal method is warranted.

## Conflict of Interest Statement

The authors declare that the research was conducted in the absence of any commercial or financial relationships that could be construed as a potential conflict of interest.

## Supplementary Material

The Supplementary Material for this article can be found online at http://www.frontiersin.org/Journal/10.3389/fmed.2014.00057/abstract
