# Supplementary material for: Perturbations in the Urinary Exosome in Transplant Rejection
Source: Front Med (Lausanne). 2015 Jan 5;1:57. doi: 10.3389/fmed.2014.00057 (PMC4292055; doi:10.3389/fmed.2014.00057)
Supplement: Supplementary file 1 [file Data_Sheet_1.DOCX]

Supplemental Table 1

| **S.No.** | **Protein name** | **Protein UniProt ID** |
| --- | --- | --- |
| 1 | Bromodomain and WD repeat-containing protein 3 | BRWD3_HUMAN |
| 2 | ProSAAS | PCSK1_HUMAN |
| 3 | C-type lectin domain family 14 member A | CLC14_HUMAN |
| 4 | Attractin | ATRN_HUMAN |
| 5 | Peptidyl-prolyl cis-trans isomerase A | PPIA_HUMAN |
| 6 | Aminopeptidase N | AMPN_HUMAN |
| 7 | Hepatitis A virus cellular receptor 2 | TIMD3_HUMAN |
| 8 | Cathepsin G | CATG_HUMAN |
| 9 | ADP-ribosyl cyclase 2 | BST1_HUMAN |
| 10 | Tubulin beta-2A chain | TBB2A_HUMAN |
| 11 | Seizure 6-like protein 2 | SE6L2_HUMAN |
| 12 | Trefoil factor 1 | TFF1_HUMAN |
| 13 | Meprin A subunit beta | MEP1B_HUMAN |
| 14 | Cyclic AMP-responsive element-binding protein 3-like protein 3 | CR3L3_HUMAN |
| 15 | Ig heavy chain V-III region VH26 | HV303_HUMAN |
| 16 | Proactivator polypeptide | SAP_HUMAN |
| 17 | Complement factor H-related protein 3 | FHR3_HUMAN |
| 18 | Ig kappa chain V-III region SIE | KV302_HUMAN |
| 19 | Ig heavy chain V-III region BRO | HV305_HUMAN |
| 20 | Complement factor D | CFAD_HUMAN |
| 21 | Ig kappa chain V-III region POM | KV306_HUMAN |
| 22 | Ig alpha-2 chain C region | IGHA2_HUMAN |
| 23 | Cell surface glycoprotein MUC18 | MUC18_HUMAN |
| 24 | Thrombospondin-1 | TSP1_HUMAN |
| 25 | Zinc finger protein 317 | ZN317_HUMAN |
| 26 | Macrophage colony-stimulating factor 1 | CSF1_HUMAN |
| 27 | Phosphoinositide-3-kinase-interacting protein 1 | P3IP1_HUMAN |
| 28 | Out at first protein homolog | OAF_HUMAN |
| 29 | Amyloid beta A4 protein | A4_HUMAN |
| 30 | Phosphatidylethanolamine-binding protein 4 | PEBP4_HUMAN |
| 31 | Histone H2A type 1-B/E | H2A1B_HUMAN |
| 32 | Dermatopontin | DERM_HUMAN |
| 33 | Protein NOV homolog | NOV_HUMAN |
| 34 | Myeloperoxidase | PERM_HUMAN |
| 35 | Glutamine-rich protein 2 | QRIC2_HUMAN |
| 36 | Cathelicidin antimicrobial peptide | CAMP_HUMAN |
| 37 | Synapsin-2 | SYN2_HUMAN |
| 38 | Kallikrein-1 | KLK1_HUMAN |
| 39 | Protein-glutamine gamma-glutamyltransferase E | TGM3_HUMAN |
| 40 | Ig kappa chain V-III region VG | KV309_HUMAN |
| 41 | CMRF35-like molecule 9 | CLM9_HUMAN |
| 42 | Cytochrome c | CYC_HUMAN |
| 43 | Putative lipocalin 1-like protein 1 | LC1L1_HUMAN |
| 44 | Tissue factor pathway inhibitor | TFPI1_HUMAN |
| 45 | Signal-regulatory protein beta-1 | SIRB1_HUMAN |
| 46 | Complement C2 | CO2_HUMAN |
| 47 | Ig lambda chain C regions | LAC_HUMAN |
| 48 | Insulin-like growth factor-binding protein 7 | IBP7_HUMAN |
| 49 | Granulins | GRN_HUMAN |
| 50 | Low affinity immunoglobulin gamma Fc region receptor III-A | FCG3A_HUMAN |
| 51 | Tubulin beta-2C chain | TBB2C_HUMAN |
| 52 | Ig kappa chain V-I region AG | KV101_HUMAN |
| 53 | Apolipoprotein F | APOF_HUMAN |
| 54 | Gelsolin | GELS_HUMAN |
| 55 | Interleukin-6 receptor subunit alpha | IL6RA_HUMAN |
| 56 | Fibrillin-1 | FBN1_HUMAN |
| 57 | Multimerin-2 | MMRN2_HUMAN |
| 58 | Endosialin | CD248_HUMAN |
| 59 | Actin, aortic smooth muscle | ACTA_HUMAN |
| 60 | Insulin-like growth factor II | IGF2_HUMAN |
| 61 | Extracellular matrix protein 1 | ECM1_HUMAN |
| 62 | Ig kappa chain V-III region CLL | KV308_HUMAN |
| 63 | Opioid-binding protein/cell adhesion molecule | OPCM_HUMAN |
| 64 | Tubulin alpha-4A chain | TBA4A_HUMAN |
| 65 | Axin-1 | AXN1_HUMAN |
| 66 | Ig gamma-2 chain C region | IGHG2_HUMAN |
| 67 | Complement component C8 gamma chain | CO8G_HUMAN |
| 68 | Plastin-2 | PLSL_HUMAN |
| 69 | Hemoglobin subunit zeta | HBAZ_HUMAN |
| 70 | Ig lambda chain V-III region SH | LV301_HUMAN |
| 71 | Non-secretory ribonuclease | RNAS2_HUMAN |
| 72 | Alpha-2-HS-glycoprotein | FETUA_HUMAN |
| 73 | Tubulin alpha-1A chain | TBA1A_HUMAN |
| 74 | Beta-2-glycoprotein 1 | APOH_HUMAN |
| 75 | Keratin, type II cytoskeletal 2 epidermal | K22E_HUMAN |
| 76 | Ig lambda-6 chain C region | LAC6_HUMAN |
| 77 | Beta-glucuronidase | BGLR_HUMAN |
| 78 | Histone H2A type 1-A | H2A1A_HUMAN |
| 79 | Resistin | RETN_HUMAN |
| 80 | N-acetylmuramoyl-L-alanine amidase | PGRP2_HUMAN |
| 81 | Alpha-amylase 1 | AMY1_HUMAN |
| 82 | Ig kappa chain V-I region EU | KV106_HUMAN |
| 83 | Ephrin type-A receptor 1 | EPHA1_HUMAN |
| 84 | L-selectin | LYAM1_HUMAN |
| 85 | Histone H2B type 1-A | H2B1A_HUMAN |
| 86 | Vitronectin | VTNC_HUMAN |
| 87 | Limbic system-associated membrane protein | LSAMP_HUMAN |
| 88 | Putative alpha-1-antitrypsin-related protein | A1ATR_HUMAN |
| 89 | Tubulin-specific chaperone E | TBCE_HUMAN |
| 90 | Tyrosine-protein kinase receptor UFO | UFO_HUMAN |
| 91 | Inter-alpha-trypsin inhibitor heavy chain H4 | ITIH4_HUMAN |
| 92 | Serum amyloid P-component | SAMP_HUMAN |
| 93 | Phosphoglycerate kinase 1 | PGK1_HUMAN |
| 94 | WAP four-disulfide core domain protein 2 | WFDC2_HUMAN |
| 95 | Neuronal growth regulator 1 | NEGR1_HUMAN |
| 96 | Osteoclast-associated immunoglobulin-like receptor | OSCAR_HUMAN |
| 97 | Keratin, type I cytoskeletal 9 | K1C9_HUMAN |
| 98 | Matrix-remodeling-associated protein 8 | MXRA8_HUMAN |
| 99 | Retinol-binding protein 4 | RET4_HUMAN |
| 100 | Fibronectin | FINC_HUMAN |
| 101 | Liver carboxylesterase 1 | EST1_HUMAN |
| 102 | Tetranectin | TETN_HUMAN |
| 103 | Sushi domain-containing protein 2 | SUSD2_HUMAN |
| 104 | Fibulin-1 | FBLN1_HUMAN |
| 105 | Complement C4-A | CO4A_HUMAN |
| 106 | T-cell antigen CD7 | CD7_HUMAN |
| 107 | Peroxiredoxin-1 | PRDX1_HUMAN |
| 108 | Galectin-3 | LEG3_HUMAN |
| 109 | Protein CutA | CUTA_HUMAN |
| 110 | Cartilage intermediate layer protein 2 | CILP2_HUMAN |
| 111 | Insulin-like growth factor-binding protein 3 | IBP3_HUMAN |
| 112 | L-lactate dehydrogenase B chain | LDHB_HUMAN |
| 113 | Gamma-glutamyl hydrolase | GGH_HUMAN |
| 114 | Endoglin | EGLN_HUMAN |
| 115 | Cadherin-13 | CAD13_HUMAN |
| 116 | Urokinase-type plasminogen activator | UROK_HUMAN |
| 117 | Sialidase-1 | NEUR1_HUMAN |
| 118 | Protein S100-A6 | S10A6_HUMAN |
| 119 | Inter-alpha-trypsin inhibitor heavy chain H1 | ITIH1_HUMAN |
| 120 | Mucosal addressin cell adhesion molecule 1 | MADCA_HUMAN |
| 121 | Heat shock 70 kDa protein 1A/1B | HSP71_HUMAN |
| 122 | Protein AMBP | AMBP_HUMAN |
| 123 | Interleukin-18-binding protein | I18BP_HUMAN |
| 124 | Signal-regulatory protein beta-1 isoform 3 | SIRBL_HUMAN |
| 125 | Endothelial cell-selective adhesion molecule | ESAM_HUMAN |
| 126 | Plasma serine protease inhibitor | IPSP_HUMAN |
| 127 | Trinucleotide repeat-containing gene 6C protein | TNR6C_HUMAN |
| 128 | Ig heavy chain V-III region GAL | HV320_HUMAN |
| 129 | Prostatic acid phosphatase | PPAP_HUMAN |
| 130 | Pigment epithelium-derived factor | PEDF_HUMAN |
| 131 | Pappalysin-2 | PAPP2_HUMAN |
| 132 | Collagen alpha-1(XII) chain | COCA1_HUMAN |
| 133 | Immunoglobulin J chain | IGJ_HUMAN |
| 134 | Inter-alpha-trypsin inhibitor heavy chain H2 | ITIH2_HUMAN |
| 135 | Vascular cell adhesion protein 1 | VCAM1_HUMAN |
| 136 | Leukocyte-associated immunoglobulin-like receptor 1 | LAIR1_HUMAN |
| 137 | Collagen alpha-3(VI) chain | CO6A3_HUMAN |
| 138 | Complement component C1q receptor | C1QR1_HUMAN |
| 139 | Angiotensinogen | ANGT_HUMAN |
| 140 | Ig kappa chain V-III region NG9 | KV303_HUMAN |
| 141 | Lysozyme C | LYSC_HUMAN |
| 142 | Transthyretin | TTHY_HUMAN |
| 143 | Superoxide dismutase [Cu-Zn] | SODC_HUMAN |
| 144 | Protein YIPF3 | YIPF3_HUMAN |
| 145 | Antithrombin-III | ANT3_HUMAN |
| 146 | Keratin, type II cytoskeletal 1 | K2C1_HUMAN |
| 147 | CD320 antigen | CD320_HUMAN |
| 148 | Annexin A2 | ANXA2_HUMAN |
| 149 | Fibulin-2 | FBLN2_HUMAN |
| 150 | Cathepsin Z | CATZ_HUMAN |
| 151 | Complement component C8 alpha chain | CO8A_HUMAN |
| 152 | Cartilage oligomeric matrix protein | COMP_HUMAN |
| 153 | Tenascin | TENA_HUMAN |
| 154 | Protein shisa-5 | SHSA5_HUMAN |
| 155 | Trefoil factor 3 | TFF3_HUMAN |
| 156 | Carboxypeptidase N catalytic chain | CBPN_HUMAN |
| 157 | Complement factor H-related protein 2 | FHR2_HUMAN |
| 158 | Mucin-5B | MUC5B_HUMAN |
| 159 | Multimerin-1 | MMRN1_HUMAN |
| 160 | Ig gamma-4 chain C region | IGHG4_HUMAN |
| 161 | Calcium-activated chloride channel regulator 1 | CLCA1_HUMAN |
| 162 | Prostaglandin-H2 D-isomerase | PTGDS_HUMAN |
| 163 | Hemoglobin subunit delta | HBD_HUMAN |
| 164 | E3 ubiquitin-protein ligase NEDD4 | NEDD4_HUMAN |
| 165 | Ig gamma-1 chain C region | IGHG1_HUMAN |
| 166 | Heat shock protein beta-1 | HSPB1_HUMAN |
| 167 | Dipeptidyl peptidase 2 | DPP2_HUMAN |
| 168 | Ephrin type-B receptor 4 | EPHB4_HUMAN |
| 169 | Bone marrow proteoglycan | PRG2_HUMAN |
| 170 | Neutrophil defensin 1 | DEF1_HUMAN |
| 171 | Extracellular superoxide dismutase [Cu-Zn] | SODE_HUMAN |
| 172 | Nicotinate-nucleotide pyrophosphorylase [carboxylating] | NADC_HUMAN |
| 173 | Keratin, type II cytoskeletal 5 | K2C5_HUMAN |
| 174 | Chondroitin sulfate proteoglycan 4 | CSPG4_HUMAN |
| 175 | Insulin-like growth factor-binding protein complex acid labile subunit | ALS_HUMAN |
| 176 | Ig gamma-3 chain C region | IGHG3_HUMAN |
| 177 | Keratin, type I cytoskeletal 10 | K1C10_HUMAN |
| 178 | Vitelline membrane outer layer protein 1 homolog | VMO1_HUMAN |
| 179 | Keratin, type I cytoskeletal 14 | K1C14_HUMAN |
| 180 | Alpha-1B-glycoprotein | A1BG_HUMAN |
| 181 | Ig kappa chain V-IV region | KV401_HUMAN |
| 182 | SH3 domain-binding glutamic acid-rich-like protein 3 | SH3L3_HUMAN |
| 183 | Ig alpha-1 chain C region | IGHA1_HUMAN |
| 184 | Ferritin light chain | FRIL_HUMAN |
| 185 | Beta-2-microglobulin | B2MG_HUMAN |
| 186 | Pro-epidermal growth factor | EGF_HUMAN |
| 187 | Lactotransferrin | TRFL_HUMAN |
| 188 | Protein S100-A9 | S10A9_HUMAN |
| 189 | Trefoil factor 2 | TFF2_HUMAN |
| 190 | Inter-alpha-trypsin inhibitor heavy chain H3 | ITIH3_HUMAN |
| 191 | V-set and immunoglobulin domain-containing protein 4 | VSIG4_HUMAN |
| 192 | ATP-binding cassette sub-family B member 9 | ABCB9_HUMAN |
| 193 | Guanylin | GUC2A_HUMAN |
| 194 | Tubulointerstitial nephritis antigen-like | TINAL_HUMAN |
| 195 | Beta-Ala-His dipeptidase | CNDP1_HUMAN |
| 196 | Cystatin-C | CYTC_HUMAN |
| 197 | Kininogen-1 | KNG1_HUMAN |
| 198 | Sialate O-acetylesterase | SIAE_HUMAN |
| 199 | BAH and coiled-coil domain-containing protein 1 | BAHC1_HUMAN |
| 200 | Maltase-glucoamylase, intestinal | MGA_HUMAN |
| 201 | Myosin-IXa | MYO9A_HUMAN |
| 202 | Ig kappa chain C region | IGKC_HUMAN |
| 203 | Prothrombin | THRB_HUMAN |
| 204 | Complement decay-accelerating factor | DAF_HUMAN |
| 205 | Prostasin | PRSS8_HUMAN |
| 206 | Plasma protease C1 inhibitor | IC1_HUMAN |
| 207 | Ig kappa chain V-I region HK102 | KV110_HUMAN |
| 208 | Vasorin | VASN_HUMAN |
| 209 | Protein HEG homolog 1 | HEG1_HUMAN |
| 210 | Ig kappa chain V-III region B6 | KV301_HUMAN |
| 211 | V-type proton ATPase 116 kDa subunit a isoform 1 | VPP1_HUMAN |
| 212 | Cubilin | CUBN_HUMAN |
| 213 | N-acetylglucosamine-6-sulfatase | GNS_HUMAN |
| 214 | Monocyte differentiation antigen CD14 | CD14_HUMAN |
| 215 | Glutathione peroxidase 3 | GPX3_HUMAN |
| 216 | Galectin-3-binding protein | LG3BP_HUMAN |
| 217 | Osteopontin | OSTP_HUMAN |
| 218 | Tyrosine-protein phosphatase non-receptor type substrate 1 | SHPS1_HUMAN |
| 219 | Gamma-glutamyltranspeptidase 1 | GGT1_HUMAN |
| 220 | Keratin, type II cytoskeletal 6A | K2C6A_HUMAN |
| 221 | Butyrophilin subfamily 2 member A1 | BT2A1_HUMAN |
| 222 | Apolipoprotein E | APOE_HUMAN |
| 223 | Hyaluronan-binding protein 2 | HABP2_HUMAN |
| 224 | Basement membrane-specific heparan sulfate proteoglycan core protein | PGBM_HUMAN |
| 225 | Apolipoprotein M | APOM_HUMAN |
| 226 | Retinoic acid receptor responder protein 1 | TIG1_HUMAN |
| 227 | Insulin-like growth factor-binding protein 6 | IBP6_HUMAN |
| 228 | Polymeric immunoglobulin receptor | PIGR_HUMAN |
| 229 | Lithostathine-1-alpha | REG1A_HUMAN |
| 230 | Peptidase inhibitor 16 | PI16_HUMAN |
| 231 | Plasminogen | PLMN_HUMAN |
| 232 | Dipeptidyl peptidase 1 | CATC_HUMAN |
| 233 | Complement C3 | CO3_HUMAN |
| 234 | Complement factor B | CFAB_HUMAN |
| 235 | Collagen alpha-1(VI) chain | CO6A1_HUMAN |
| 236 | Nidogen-1 | NID1_HUMAN |
| 237 | Complement component C6 | CO6_HUMAN |
| 238 | Coagulation factor XII | FA12_HUMAN |
| 239 | Serotransferrin | TRFE_HUMAN |
| 240 | Ig lambda chain V region 4A | LV001_HUMAN |
| 241 | Dipeptidyl peptidase 3 | DPP3_HUMAN |
| 242 | Multidrug resistance-associated protein 7 | MRP7_HUMAN |
| 243 | Alpha-2-macroglobulin | A2MG_HUMAN |
| 244 | EGF-containing fibulin-like extracellular matrix protein 1 | FBLN3_HUMAN |
| 245 | Corticosteroid-binding globulin | CBG_HUMAN |
| 246 | Myotubularin-related protein 5 | MTMR5_HUMAN |
| 247 | Complement factor H | CFAH_HUMAN |
| 248 | CD59 glycoprotein | CD59_HUMAN |
| 249 | Ig heavy chain V-III region WEA | HV302_HUMAN |
| 250 | Serum paraoxonase/lactonase 3 | PON3_HUMAN |
| 251 | Lysosomal acid phosphatase | PPAL_HUMAN |
| 252 | Zinc-alpha-2-glycoprotein | ZA2G_HUMAN |
| 253 | Endonuclease domain-containing 1 protein | ENDD1_HUMAN |
| 254 | Plasma kallikrein | KLKB1_HUMAN |
| 255 | Serum amyloid A-4 protein | SAA4_HUMAN |
| 256 | Pancreatic secretory trypsin inhibitor | ISK1_HUMAN |
| 257 | Secreted Ly-6/uPAR-related protein 1 | SLUR1_HUMAN |
| 258 | Poliovirus receptor-related protein 2 | PVRL2_HUMAN |
| 259 | Fibrinogen gamma chain | FIBG_HUMAN |
| 260 | Osteomodulin | OMD_HUMAN |
| 261 | Poliovirus receptor | PVR_HUMAN |
| 262 | Plasma glutamate carboxypeptidase | PGCP_HUMAN |
| 263 | ICOS ligand | ICOSL_HUMAN |
| 264 | Arylsulfatase A | ARSA_HUMAN |
| 265 | Heat shock protein HSP 90-alpha | HS90A_HUMAN |
| 266 | Hemoglobin subunit beta | HBB_HUMAN |
| 267 | Serum albumin | ALBU_HUMAN |
| 268 | Complement factor I | CFAI_HUMAN |
| 269 | CD44 antigen | CD44_HUMAN |
| 270 | Alpha-1-antichymotrypsin | AACT_HUMAN |
| 271 | Histidine-rich glycoprotein | HRG_HUMAN |
| 272 | Ceruloplasmin | CERU_HUMAN |
| 273 | Collagen alpha-2(V) chain | CO5A2_HUMAN |
| 274 | Probable G-protein coupled receptor 113 | GP113_HUMAN |
| 275 | Serpin B3 | SPB3_HUMAN |
| 276 | Uromodulin | UROM_HUMAN |
| 277 | Putative uncharacterized protein UNQ6975/PRO21958 | YB002_HUMAN |
| 278 | Gamma-interferon-inducible lysosomal thiol reductase | GILT_HUMAN |
| 279 | Complement component C7 | CO7_HUMAN |
| 280 | Plasminogen-related protein A | PLGA_HUMAN |
| 281 | Charged multivesicular body protein 1b | CHM1B_HUMAN |
| 282 | Alpha-1-acid glycoprotein 1 | A1AG1_HUMAN |
| 283 | Moesin | MOES_HUMAN |
| 284 | Lumican | LUM_HUMAN |
| 285 | Leucine-rich alpha-2-glycoprotein | A2GL_HUMAN |
| 286 | Hemicentin-1 | HMCN1_HUMAN |
| 287 | Apolipoprotein A-IV | APOA4_HUMAN |
| 288 | Hemopexin | HEMO_HUMAN |
| 289 | Complement component C9 | CO9_HUMAN |
| 290 | Serum paraoxonase/arylesterase 1 | PON1_HUMAN |
| 291 | Apolipoprotein C-II | APOC2_HUMAN |
| 292 | Apolipoprotein B-100 | APOB_HUMAN |
| 293 | Heat shock-related 70 kDa protein 2 | HSP72_HUMAN |
| 294 | Fibrinogen alpha chain | FIBA_HUMAN |
| 295 | Thyroxine-binding globulin | THBG_HUMAN |
| 296 | Tripeptidyl-peptidase 1 | TPP1_HUMAN |
| 297 | Calbindin | CALB1_HUMAN |
| 298 | Prostate stem cell antigen | PSCA_HUMAN |
| 299 | Nuclear transport factor 2 | NTF2_HUMAN |
| 300 | Fibrinogen beta chain | FIBB_HUMAN |
| 301 | Vitamin D-binding protein | VTDB_HUMAN |
| 302 | Low-density lipoprotein receptor-related protein 2 | LRP2_HUMAN |
| 303 | Complement C1s subcomponent | C1S_HUMAN |
| 304 | Hemoglobin subunit alpha | HBA_HUMAN |
| 305 | Apolipoprotein(a) | APOA_HUMAN |
| 306 | Thioredoxin | THIO_HUMAN |
| 307 | Clusterin | CLUS_HUMAN |
| 308 | Cadherin-1 | CADH1_HUMAN |
| 309 | Cathepsin D | CATD_HUMAN |
| 310 | Catalase | CATA_HUMAN |
| 311 | Protein disulfide-isomerase | PDIA1_HUMAN |
| 312 | C4b-binding protein alpha chain | C4BPA_HUMAN |
| 313 | Alpha-1-antitrypsin | A1AT_HUMAN |
| 314 | Phosphoglycerate mutase 1 | PGAM1_HUMAN |
| 315 | Roundabout homolog 4 | ROBO4_HUMAN |
| 316 | Afamin | AFAM_HUMAN |
| 317 | Twisted gastrulation protein homolog 1 | TWSG1_HUMAN |
| 318 | Mannan-binding lectin serine protease 2 | MASP2_HUMAN |
| 319 | Apolipoprotein A-I | APOA1_HUMAN |
| 320 | Vitamin K-dependent protein S | PROS_HUMAN |
| 321 | HLA class I histocompatibility antigen, A-23 alpha chain | 1A23_HUMAN |
| 322 | Vesicular integral-membrane protein VIP36 | LMAN2_HUMAN |
| 323 | Endothelial protein C receptor | EPCR_HUMAN |
| 324 | Lymphatic vessel endothelial hyaluronic acid receptor 1 | LYVE1_HUMAN |
| 325 | CD5 antigen-like | CD5L_HUMAN |
| 326 | Complement C1r subcomponent-like protein | C1RL_HUMAN |
| 327 | Apolipoprotein D | APOD_HUMAN |
| 328 | Cystatin-M | CYTM_HUMAN |
| 329 | Glutamyl aminopeptidase | AMPE_HUMAN |
| 330 | Alpha-1-acid glycoprotein 2 | A1AG2_HUMAN |
| 331 | Uteroglobin | UTER_HUMAN |
| 332 | Sushi domain-containing protein 5 | SUSD5_HUMAN |
| 333 | Haptoglobin | HPT_HUMAN |
| 334 | Ig mu chain C region | IGHM_HUMAN |
| 335 | Complement C1q subcomponent subunit B | C1QB_HUMAN |
| 336 | Myelin basic protein | MBP_HUMAN |
| 337 | Mucin-1 | MUC1_HUMAN |
| 338 | Protein S100-A8 | S10A8_HUMAN |
| 339 | Defensin-5 | DEF5_HUMAN |
| 340 | Procollagen C-endopeptidase enhancer 1 | PCOC1_HUMAN |
| 341 | Haptoglobin-related protein | HPTR_HUMAN |
| 342 | Immunoglobulin superfamily containing leucine-rich repeat protein | ISLR_HUMAN |
| 343 | Apolipoprotein A-II | APOA2_HUMAN |
| 344 | Triosephosphate isomerase | TPIS_HUMAN |
| 345 | Complement C1r subcomponent | C1R_HUMAN |
| 346 | Uncharacterized protein KIAA0753 | K0753_HUMAN |
| 347 | Immunoglobulin lambda-like polypeptide 1 | IGLL1_HUMAN |
| 348 | Coiled-coil domain-containing protein 65 | CCD65_HUMAN |
| 349 | Ribonuclease pancreatic | RNAS1_HUMAN |
